# Shape-Dependent Single-Electron Levels for Au Nanoparticles

**DOI:** 10.3390/ma9040301

**Published:** 2016-04-21

**Authors:** Georgios D. Barmparis, Georgios Kopidakis, Ioannis N. Remediakis

**Affiliations:** 1 Department of Materials Science and Technology, University of Crete, 71003 Heraklion, Greece; barmparis@physics.uoc.gr (G.D.B.); kopidaki@materials.uoc.gr (G.K.); 2 Crete Center for Quantum Complexity and Nanotechnology, Department of Physics, University of Crete, 71003 Heraklion, Greece; 3 Institute of Electronic Structure and Laser, Foundation for Research and Technology-Hellas, 70013 Heraklion, Greece

**Keywords:** material design, gold, nanoparticles, nanomaterials, single-electron states

## Abstract

The shape of metal nanoparticles has a crucial role in their performance in heterogeneous catalysis as well as photocatalysis. We propose a method of determining the shape of nanoparticles based on measurements of single-electron quantum levels. We first consider nanoparticles in two shapes of high symmetry: cube and sphere. We then focus on Au nanoparticles in three characteristic shapes that can be found in metal/inorganic or metal/organic compounds routinely used in catalysis and photocatalysis. We describe the methodology we use to solve the Schrödinger equation for arbitrary nanoparticle shape. The method gives results that agree well with analytical solutions for the high-symmetry shapes. When we apply our method in realistic gold nanoparticle models, which are obtained from Wulff construction based on first principles calculations, the single-electron levels and their density of states exhibit distinct shape-dependent features. Results for clean-surface nanoparticles are closer to those for cubic particles, while CO-covered nanoparticles have energy levels close to those of a sphere. Thiolate-covered nanoparticles with multifaceted polyhedral shape have distinct levels that are in between those for sphere and cube. We discuss how shape-dependent electronic structure features could be identified in experiments and thus guide catalyst design.

## 1. Introduction

Although most properties of nanoparticle-containing materials are governed by nanoparticle size, the importance of nanoparticle shape is increasingly appreciated for applications, most notably in optoelectronics and catalysis. Consequently, in addition to controlling size, considerable effort has recently been devoted to determining and tailoring the exact atomic arrangement in nanoparticles. The shape of nanoparticles plays a crucial role in the efficiency of many types of catalysts, such as transition metal heterogeneous catalysts [1,2,3] and titanium oxide photocatalysts [4,5]. For example, for rutile-supported Au, the supporting oxide is found to enhance the catalytic properties of the metal by enforcing specific shapes on the metal clusters, and also by providing a reaction path at the metal oxide interface [6]. The shape of Au nanoparticles can be easily modified during synthesis by means of surfactants and other experimental conditions [7,8,9,10].

The precise characterization of the shape of a metal nanoparticle in a sample is not a trivial task. Despite enormous progress and widespread use during the last few decades, atomic-scale imaging techniques now described in textbooks [11], such as optical, electron, scanning tunneling and atomic force microspcopy, present technical difficulties and inherent resolution limitations. Accurate atomic-level nanostructure determination requires state-of the-art methods which often combine experimental techniques and nanoparticle modeling [12,13]. On the other hand, the quantum energies in metal nanoparticles are clearly affected by their shape [14] and the presence of adsorbates, as shown both theoretically [15] and experimentally [16]. Here we propose an alternative method of shape characterization: probing the single-electron energy levels of the nanoparticles.

In addition to shape-probing, the study of electron confinement in nanoparticles has applications in many other fields of modern materials science. Quantization phenomena are important even in large nanoparticles, where single-electron discrete energy level spacing is on the order of a few meV. There are optical and electrical properties which are determined by quantized electronic states and can be used to probe nanoparticle size and shape. Optical measurements, such as absorption spectra, that provide discrete levels for semiconductor nanoparticles, might be difficult to apply to metal nanoparticles, where plasmon resonance effects dominate in optical frequencies [17,18]. This is especially the case with gold, where bulk plasmon frequencies in the UV shift in the range of visible light for nanoparticles. Interaction of metal nanoparticles with light depends on their composition, size, and shape, as well as on the surrounding medium. Extensive work over the past century, starting with light scattering and absorption from spherical particles [19] and developed in the field of plasmonics, has led to the complete understanding of light nanoparticle interaction and to diverse practical applications [20]. Resonances, local-field enhancement, and absorption are used in optoelectronic devices [21,22]; in photovoltaics, photo-sensitive materials include nanoparticles tuned so that the peak of their absorption coefficient is close to the maximum of the solar spectrum [23]; in the paint industry, nanoparticles give specific colors to colloidal suspensions [24]. Theoretical results connect optical data with the size and the overall nanoparticle shape characteristics [25,26], but not with the details of the atomic arrangement. Electrical measurements are another possibility for investigating metal nanoparticle properties. Discrete electronic levels are probed by single-electron tunneling spectroscopy with single electron transistors (SET) [27,28,29,30].

In this work, we present results for the electronic structure of realistic gold nanoparticles. The nanoparticle models were obtained by an atomistic Wulff construction in inert and reactive environment using density functional theory (DFT) results for the surface energies of different facets. The shape of metal nanoparticles at equilibrium depends on the surface energies of the various (hkl) crystalline planes of the material. Strong interactions between the metallic particle and the surrounding inorganic or organic matter result in significant changes of its shape, which in turn modifies its electronic, optical, and catalytic properties. We show how discrete energy levels and the corresponding density of states are related to nanoparticle shape so that knowledge about nanoparticle electronic structure can be used for shape determination. SET experiments with gold nanoparticles indicate that the independent electron approximation is valid [28]. The eigenenergies of the Schrödinger equation for a free electron moving inside the nanoparticle will be dependent on the nanoparticle shape. Reversing the problem, if one measures a series of electron energies for a given material, they can deduce what nanoparticle shape corresponds to this distribution of energies.

The task in front of us is similar to the problem set by mathematician Mark Kac fifty years ago: Can one hear the shape of a drum [31,32]? In other words, can one deduce the shape of a two-dimensional drum from the vibrational eigenvalues of its membrane? The Schrödinger equation for a single free electron in a nanoparticle is mathematically the same as the Helmholtz equation that gives the eigenfrequencies of vibration, the only difference being the number of dimensions (three *vs.* two). There is no clear answer to this question in three dimensions, although this is still an active research topic—see reference [33] for another application in nanoparticles.

## 2. Results

### 2.1. Single-Electron Levels for Typical Nanoparticle Shapes

The task before us is to find stationary states of an electron that is free to move inside a nanoparticle. No force is acting on the electron, and the only constraint in its motion is that it cannot escape the confinement space, and therefore its wavefunction will be zero along the planes that define the boundary of the nanoparticle. The energy levels corresponding to stationary states for this free electron can be found by solving the time-independent Schrödinger equation:

(1) $$\left[-\frac{\hbar^{2}}{2m}\nabla^{2}+V\left(x,y,z\right)\right]\psi_{i}\left(x,y,z\right)=E_{i}\psi_{i}\left(x,y,z\right)$$

where $E_{i}$ and $\psi_{i}\left(x,y,z\right)$ are the $i^{th}$ eigenvalue and eigenstate of the system, respectively; $-\frac{\hbar^{2}}{2m}\nabla^{2}$ is the kinetic energy of a particle with mass *m*; and V(x,y,z) is the potential energy. In our case, potential energy is defined as:

(2) $$V\left(x,y,z\right)=\left\{\begin{array}{cc} 0 & \text{inside}\;\text{the}\;\text{nanoparticle}\\ \infty & \text{otherwise} \end{array}\right.$$

The potential described in Equation (2) is the simplest possible confining potential for a metal nanoparticle. In reality, the potential will have a finite depth, which will be of the order of the work functions of the various (hkl) surfaces contained in the nanoparticle. This workfunction will in turn be modified by the presence of adsorbates, as adsorbates will modify the electronic structure, in particular for smaller nanoparticles [16]. The presence of the support might result in modifications of the nanoparticle shape [34]; charge transfer to Au nanoparticles from oxides such as rutile TiO${}_{2}$ is minimal [35]. Moreover, a realistic nanoparticle will contain structural and chemical defects that will also affect its electronic structure. All these considerations make the problem extremely complicated (and at the same time interesting), making it very difficult to study.

We use a value of 100 a.u. (about 2700 eV) as an approximation of infinity in Equation (2). We tried different values of the confining potential, but found no qualitative difference in results, such as symmetry and degeneracy of the eigenstates or the shape-dependence of the excitation energies. For this reason, we proceed with the simplest possible potential that guarantees that the wave functions are zero outside the nanoparticle. The infinite-potential well of Equation (2) is the simplest possible mathematical model that reproduces the shape-dependence of the excitation energies for nanoparticles of the same volume and the same symmetry point group.

In Section 3, we present the methodology we followed to solve Equation (1). In the following subsections, we describe our findings for five different nanoparticle shapes:

1. Cube;
2. Sphere;
3. Truncated octahedron, formed when Au has very weak interactions with its environment, such as Au nanoparticles bonded to C nanotubes [36];
4. Complex rounded shape, formed when Au is in equilibrium with small molecules with strong binding to Au, such as CO [37];
5. Deltoidal icositetrahedron, formed when Au is in equilibrium with large molecules with strong binding to Au, such as alkanethiols [15,38].

The first shape is used as the basis for all other calculations, whereas the second one is used to verify the accuracy of our numerical method. The last three shapes are typical equilibrium shapes for Au nanoparticles.

We only consider shapes that can be formed from face-centered cubic (fcc) Au under thermodynamic equilibrium, as these are the shapes most commonly seen in experiments. Shapes considered here are invariant under all transformations of the fcc lattice space group, $O_{h}$. The effect of major shape changes, such as from spheres to ellipsoids, to electron and plasmon energies is reviewed in reference [14].

#### 2.1.1. Cubic Nanoparticle

The solution of the three-dimensional “particle-in-a-box” problem with cubic symmetry can be found in any Quantum-Mechanics textbook. The eigefunctions of the Hamiltonian are

(3) $$\phi_{n,m,l}\left(x,y,z\right)=\sqrt{\frac{8}{a^{3}}}\sin\left(\frac{n\pi x}{a}\right)\sin\left(\frac{m\pi y}{a}\right)\sin\left(\frac{l\pi z}{a}\right)$$

where *a* is the length of the cube; and n,m, and *l* are positive integers, the so-called “quantum numbers” of the problem. Being eigenfunctions of a hermitian operator, the $\phi_{n,m,l}\left(x,y,z\right)$ functions form a complete and orthonormal set of functions that are vanishing in the borders of the box. The eigenvalues of the Hamiltonian, $E_{n,m,l}$, are given by:

(4) $$E_{nml}=\frac{\hbar^{2}\pi^{2}}{2m_{e}a^{2}}\left(n^{2}+m^{2}+l^{2}\right)$$

where, *ℏ* is the reduced Planck constant; and $m_{e}$ is the mass of the electron.

The ground state is non-degenerate (all three quantum numbers are equal to one). The next three eigenenergies are triply degenerate. These three triplet states have quantum numbers equal to (n,l,m)=(1,1,2),(1,2,2),(1,1,3), respectively.

We use these eigenfunctions and eigenvalues as the basis for the solution of the Schrödinger equation in other nanoparticle shapes. The details of the calculation are given in Section 3.

#### 2.1.2. Spherical Nanoparticle

The problem of an electron that moves freely in a sphere of radius *R* can also be solved analytically. The energy levels, $E_{nl}$, are given by:

(5) $$E_{nl}=\frac{\hbar^{2}\chi_{nl}^{2}}{2m_{e}R^{2}}$$

where $\chi_{nl}$ is the $n^{th}$ root of the $l^{th}$ order spherical Bessel functions $j_{l}\left(x\right)$. The corresponding eigenfunctions are

(6) $$\psi_{nlm}\left(r,\theta ,\phi \right)\propto j_{l}\left(k_{nl}r\right)Y_{lm}\left(\theta ,\phi \right)$$

where $j_{l}\left(x\right)$ is the spherical Bessel function of order *l* and $Y_{lm}\left(\theta ,\phi \right)$ is a spherical harmonic. $k_{nl}$ is related to the energy through $E_{nl}=\frac{\hbar^{2}k_{nl}^{2}}{2m_{e}}$.

The symbols *n* and *l* represent the principle quantum number and the angular momentum quantum number of each state, respectively. As usual, the third quantum number, *m*, is smaller in magnitude than *l* and does not enter Equation (5). This is the reason that each one of the energy levels is (2l+1)-fold degenerate. In contrast to what is found in the solution of the Schrödinger equation for the hydrogen atom, here *l* is not necessarily smaller than *n*. For the spherical nanoparticle, the ground state is again non-degenerate and the first excited state is a triplet. A key difference between spherical and cubic particles occurs in the next six states: we found two triplets for the cube, whereas a quintuplet and a singlet were found for the sphere.

To validate our methodology, we compare the energy levels from Equation (5) to those found by numerical solution of an atomistic model for a spherical nanoparticle. We constructed a spherical particle of volume *V* = 481.42 nm${}^{3}$ and sphericity equal to 99.6%. The sphericity of a particle is a measure of how close its shape is to a sphere. It is defined as $\pi^{1/3}{\left(6V\right)}^{2/3}/A$, where *V* is the volume and *A* is the area. A sphere has sphericity 1 while a cube has sphericity of about 0.8.

The numerical values are calculated using a set of 32,768 basis functions, with the infinite potential outside the nanoparticle defined as $V_{0}$ = 100 a.u. (*i.e.*, about 2700 eV). Both the number of basis functions and the value of $V_{0}$, are chosen so that the ground state and the 100th eigenenergy are well converged. In Table 1, we present the theoretical and calculated energies of the thirty lowest-energy eigenstates for a spherical nanoparticle with volume *V* = 481.42 nm${}^{3}$. The eigenvalues of the analytical solution for different *l* and *n* are put together and sorted; we use a new index, *i*, to count them.

The calculated eigenenergies were found to be slightly overestimated compared to the analytically obtained ones. The relative error was 4% or less. The simulated nanoparticle, being a collection of Au atoms in the fcc lattice, is not a perfect sphere; any vertices tend to increase the kinetic energy of the electron. Another factor that affects the agreement between the analytical solution and the simulation could be sought in the vague definition of “volume” for a nanoparticle. Here, for simplicity, we consider that the boundary of the nanoparticle consists of planes that pass through the nuclei of the outermost atoms. Due to the quantum nature of the electron, however, the actual volume that is available will be a bit larger. So, the simulated electron is a bit more confined and hence its energy is a bit higher.

Despite these small and expected discrepancies, overall the quality of the simulation is satisfactory: differences between energy levels are correct to the first decimal, and degeneracies are correctly reproduced. Moreover, the qualitative features of the wave functions are well represented, including the symmetries of the eigenstates.

**Figure 1:**
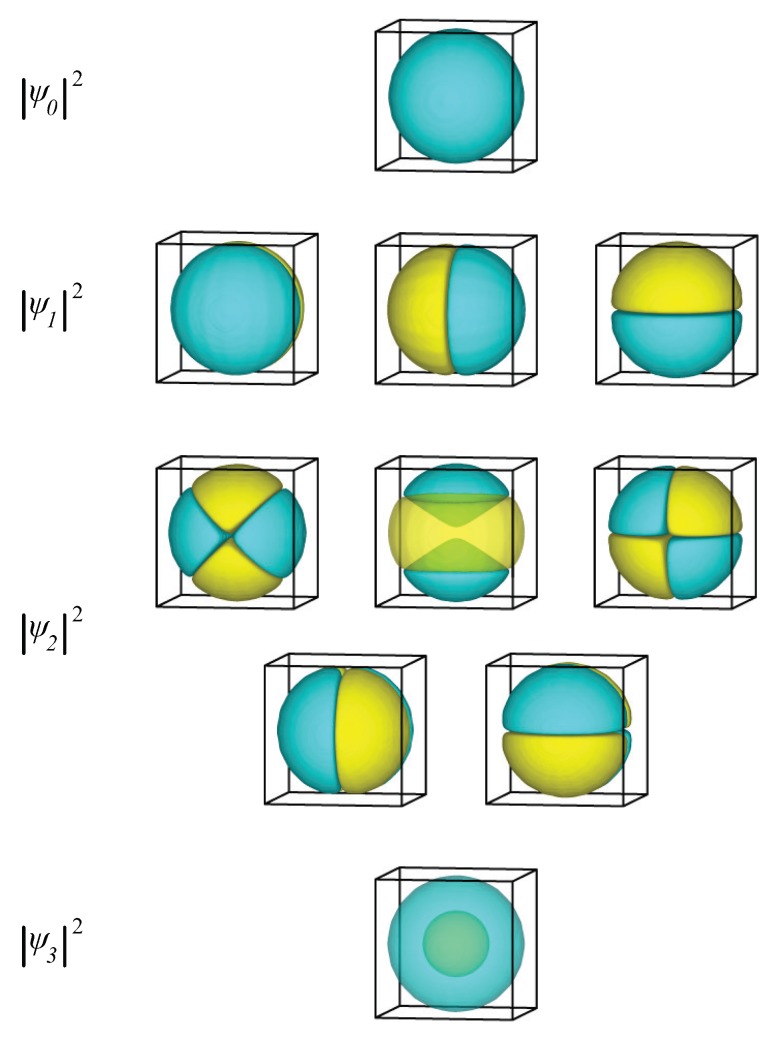
The probability function, ${\left|\psi \right|}^{2}$, of the first ten eigenstates of an electron inside a sphere. Yellow represents positive and cyan represents negative values of the wavefunction.

In Figure 1, we present the probability function, ${\left|\psi \right|}^{2}$, of the eigenfunctions for the first four calculated energy levels. In the analytical solution, the four lowest energy eigenvalues correspond to a singlet (n=1,l=0), followed by a triplet (n=1,l=1), a quintet (n=1,l=2), and a singlet (n=2,l=0). The simulations correctly yield all these degeneracies. The density plots shown in Figure 1 correctly resemble the familiar shapes of s,p,d, and $s^{*}$ orbitals, which are the real-valued linear combinations of the spherical harmonics with l=0, l=1, and l=2.

As a last test of the method, we compare the density of states per volume of an electron in a spherical nanoparticle. The density of states (DOS) per volume, $\rho_{V}\left(E\right)$, is defined as:

(7) $$\rho_{V}\left(E\right)=\frac{1}{V}\sum_{i}\delta \left(E-E_{i}\right)$$

where *V* is the volume of the nanoparticle and the sum runs over all eigenstates of the Hamiltonian. The delta function can be approximated using a Lorentzian distribution with broadening *σ*:

(8) $$\delta \left(x\right)=\frac{1}{\pi \sigma (1+\frac{x^{2}}{\sigma^{2}})}$$

**Figure 2:**
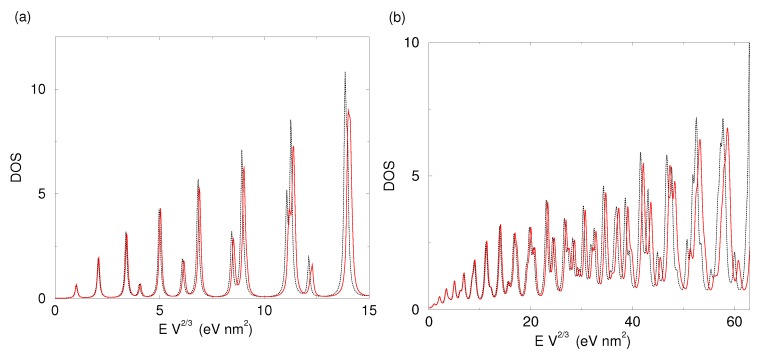
Comparison of the $\rho_{V}\left(E\right)$ of an electron in a spherical nanoparticle of volume *V* = 481.42 nm${}^{3}$ using the calculated (red line) and the analytical eigenenergies (black dotted line). Density of States (DOS) has been calculated using Lorentzian broadening of 1 meV in (**a**); and 5 meV in (**b**).

In Figure 2, we plot the $\rho_{V}\left(E\right)$ of an electron in a spherical nanoparticle for two sets of eigenenergies: those obtained from our simulation and the analytical values of them using Equation (5). We used two different broadenings for the Lorentzian approximation of the delta function in Equation (8). To make our plot size-independent, we multiply our energies with the volume to the power of 2/3.

To change our energy units to ordinary energy values, one needs to divide the values of $EV^{2/3}$ by $V^{2/3}$, that is, the volume of the nanoparticle to the power of 2/3. For example, a value of 10 eV nm${}^{2}$ in the *x*-axis of Figure 2 corresponds to 38 meV and 154 meV for a spherical nanoparticles with radii of 10 nm and 5 nm, respectively.

The calculated DOS matches the analytically obtained one very well. The first ten peaks are at the same energies, while even for quite large values the difference is small. This difference can be attributed to the underestimation of volume in the atomistic nanoparticle and is small. For instance, the value of 60 eV nm${}^{2}$, where a significant discrepancy between the two functions is observed in Figure 2b, corresponds to energy of the order of 1 eV for a nanoparticle of radius 5 nm. For comparison, the difference between the ground- and the first excited state for this nanoparticle is only 15 meV. Therefore, our method provides reliable DOS for all practical purposes.

#### 2.1.3. Single Electron in Equilibrium-Shaped Au Nanoparticles

The shape of Au nanoparticles depends on the concentration and strength of any bonds that are formed between the Au atoms at the surface of the nanoparticle and any ligands or molecules of matter that surround that nanoparticle. Formally, the shape is obtained by means of a Wulff construction where the surface tensions, *γ*, are substituted by the interfacial tensions, $\gamma_{int}$, between Au surfaces and their environment. A simple relationship between the two has been found when there is little interaction between adsorbates [39]:

(9) $$\gamma_{int}=\gamma +\theta \frac{E_{ads}}{A_{at}}$$

In this equation, *θ* is the coverage of Au surfaces (θ=1 corresponds to full coverage) while $E_{ads}$ is the adsorption energy of ligands (exothermic adsorption means negative $E_{ads}$). $A_{at}$ is the area per Au atom, that is, the total area divided by the number of surface Au atoms. According to Equation (9), $\gamma_{int}\approx \gamma$ when the coverage, *θ*, or the adsorption energy, $E_{ads}$, is small. For this reason, Au nanoparticles are found to have similar shapes in a variety of materials.

**Figure 3:**
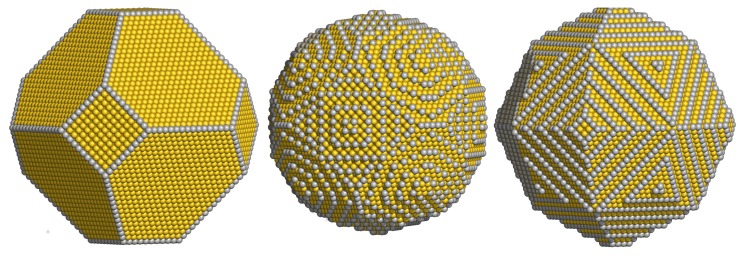
Ball-and-stick models of typical equilibrium shapes for Au nanoparticles. Step and kink atoms are shown in darker color. (**Left**): clean nanoparticle; (**center**): particle in CO-rich gas at typical CO oxidation conditions; (**right**): particle in equilibrium with thiol-containing molecules at typical polymer concentrations.

We begin by considering an atomistic Wulff construction for Au with no interactions with its environment. The same shape is found for cases where adsorbates exist on Au surfaces, but the ratio of the binding energy over the area of Au is smaller than about 5% of the surface tension of Au [39]. This shape is found to be a truncated octahedron (see Figure 3). The Wulff construction agrees very well with microscopy images of Au nanoparticles in a variety of materials [36,40,41]. In the following, we will use the term “clean” to describe this shape.

A set of 32,768 basis functions was used to calculate the eigenenergies and eigenfunctions of the nanoparticle shown in Figure 3 (left). The diameter of this nanoparticle is d=10.45 nm, it contains 28,806 atoms, its volume is 485.38 nm${}^{3}$, its sphericity equals 89.87%. The density of step-edge atoms that typically act as active sites for catalysis is 72 μmol/g.

**Figure 4:**
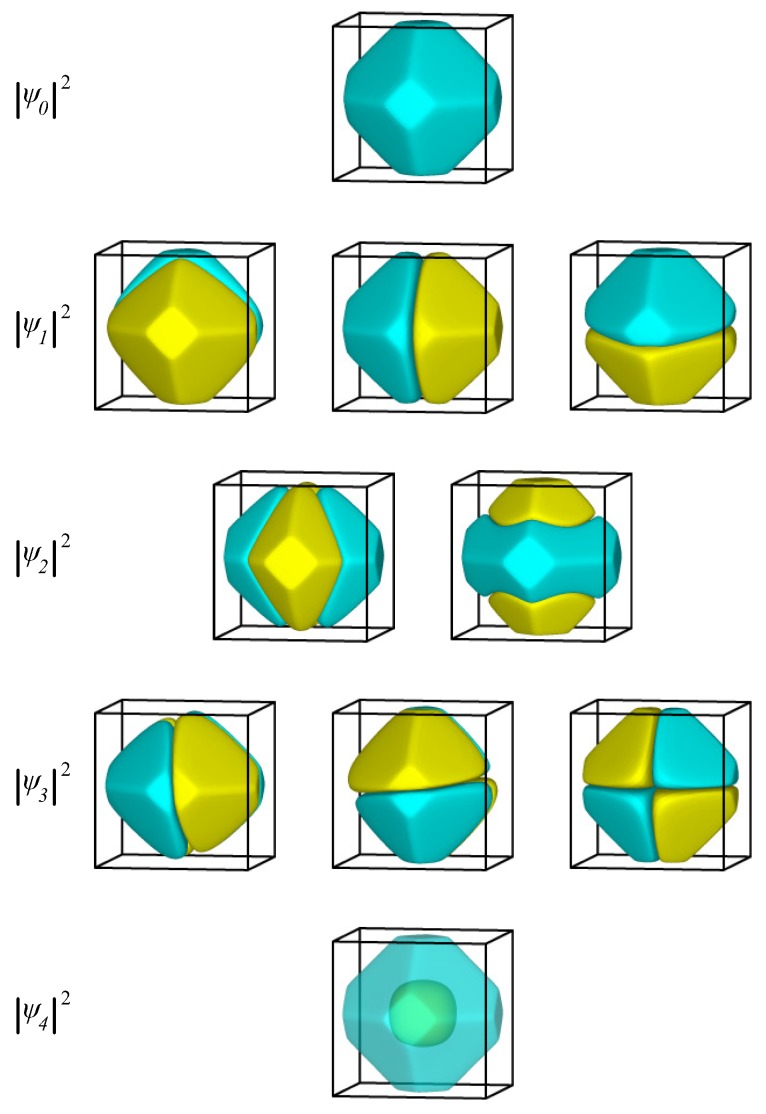
The probability function, ${\left|\psi \right|}^{2}$, of the lowest-energy eigenstates of an electron inside a clean Au nanoparticle at equilibrium. Yellow represents positive and cyan negative values of the wavefunction.

In Figure 4, we present the probability function, ${\left|\psi \right|}^{2}$, of the eigenstates for the first four eigenenergies in the case of a clean nanoparticle. Apart from the obvious change of the nanoparticle boundary, the energy degeneracy and the symmetry of the wavefunctions for the two lowest eigenenergies is the same as for the cubic nanoparticle. The ground state is non-degenerate and has an eigenfunction with the same sign everywhere. The first excited state, which is triply degenerate, has three eigenfunctions that change sign once and are antisymmetric with respect to the three main mirror planes of the nanoparticle. The next energy level, however, differs from both the cubic nanoparticle (where the third lowest eigenenergy is a triplet) and the spherical one (where the third lowest energy is a quintet). Here, due to the absence of spherical symmetry, the quintet of the l=2 states breaks into two sets: first, a doubly degenerate state with eigenfunctions similar to the $d_{z^{2}}$ orbital; second, a triply degenerate state with eigenfunctions similar to $d_{xy},d_{yz}$, and $d_{zx}$ orbitals.

#### 2.1.4. Au Nanoparticles with Strong Interactions with Their Surroundings

The shape of Au nanoparticles changes dramatically when Au has strong interactions with materials around it. However, only very strong binders and/or molecules that can bind Au at high areal densities are capable of modifying the equilibrium shape of Au particles. Here, we consider two characteristic examples: first, the shape of Au at equlibrium with CO gas at CO oxidation conditions, taken from reference [39], and second, the shape of Au at equilibrium with methanethiol molecules at a density typical for Au encapsulated in polymer matrices, taken from reference [42]. The simulated nanoparticles are shown in Figure 3.

The CO-covered one contains 27,981 atoms, its diameter is d=10.03 nm, its volume is V=500.36nm${}^{3}$, its sphericity is 98.6%, and its density of active sites is 181 μmol/g. The thiolate-covered nanoparticle has a polyhedral shape close to that of a deltoidal icositetrahedron. A particle with 28,502 atoms, d=10.87 nm, V=505.68 nm${}^{3}$, sphericity of 95.3%, and density of active sites 273 μmol/g was simulated.

**Figure 5:**
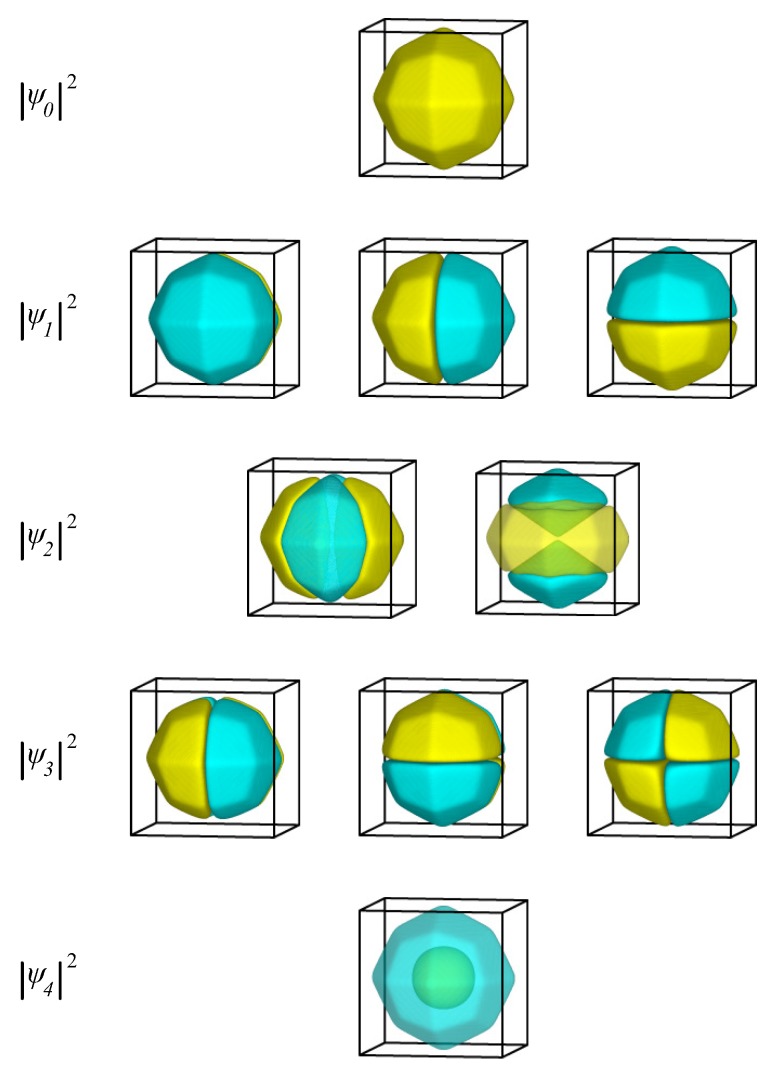
The probability function, ${\left|\psi \right|}^{2}$, of the first ten eigenstates of an electron inside a thiolate-protected Au nanoparticle. Yellow represents positive and cyan negative values of the wavefunction.

In Figure 5, we present the probability function, ${\left|\psi \right|}^{2}$, of the eigenstates for the lowest single-electron eigenenergies for the thiolate-covered gold nanoparticle. As was the case with a clean nanoparticle, the symmetry of the eigenstates lies in between the symmetry of states in a sphere and the symmetry of states in a cube. Again, the fivefold *d* state of the sphere has split into a triplet and a doublet. The main qualitative difference between protected and clean nanoparticles is that in the former, the doublet and triplet states are very close in energy, making these five states almost degenerate. The geometric characteristics of the eigenfunctions are the same as those for the clean Au nanoparticle.

### 2.2. Shape-Dependent Electronic Structure

We now compare our findings for the different nanoparticle shapes, trying to address whether is could be possible to probe the nanoparticle shape, and therefore its catalytic activity, without microscopy. In the following, we summarize the key differences in the electronic states of the shapes considered in this work and discuss possible connections to state-of-the-art experimental techniques that could probe these electronic states.

**Figure 6:**
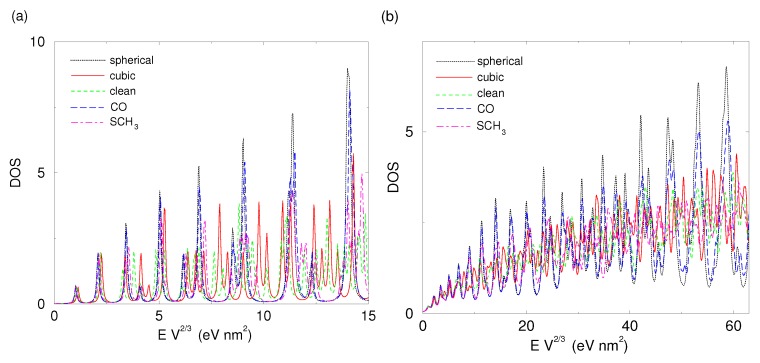
The DOS per volume of all shapes considered in this study: spherical (dotted black line), cubic (red line), clean (dashed green line), CO-covered (long-dashed blue line), and thiolate-covered (dot-dashed purple line). DOS has been calculated using Lorentzian broadening of either 1 meV (**a**) or 5 meV (**b**). The energies have been multiplied by the volume of the nanoparticle to the exponent 2/3.

In Figure 6, we present the density of states for all shapes considered in this study. In order to eliminate size-dependent properties, we use dimensional analysis to find that the density of states of a free electron is proportional to the volume while its energy is proportional to the volume to the power of $-\frac{2}{3}$. The *x*-axis can be transformed to usual energy units by multiplying with $V^{-2/3}$, where *V* is the volume of the nanoparticle; see Section 2.1.2 for examples.

Interestingly, the shape with the highest sphericity, which is the CO-covered nanoparticle, has a DOS that is very close to that of a sphere throughout the range of energies considered. The clean nanoparticle has features that resemble more those of the cube than those of the sphere. Finally, the SCH${}_{3}$-covered nanoparticle has distinct features in its DOS that make it distinguishable from both the sphere and the cube.

Let us now focus on the first few excited states and the corresponding excitation energies of the electron, which might be a more reliable probe of the shape. In Table 2 we present the ten lowest eigenvalues of the Schrödinger equation, taking the ground state energy to be zero in every case. The nanoparticles are ranked according to the first excitation energy.

A clean nanoparticle is found to have its first excitation energy 3% higher than the nanoparticles covered with CO or thiolates. Moreover, the faceted shape can be clearly differentiated from the spherical or the cubic shapes. Even the difference between CO- and thiolate-covered particles, which is only 1.9%, is undoubtedly within the resolution of modern state-of-the-art instruments.

In the second excitation energy, the differences are more prominent. A sphere has a five-fold degenerate state; cube and CO-covered nanoparticles have three-fold degenerate states; thiol-covered and clean nanoparticles have doublet states. Different degeneracies yield different peak heights in absorption experiments. For the second excitation energy, peak height might be a more important differentiation tool than peak position, as CO- and SCH${}_{3}$-covered nanoparticles have very similar energies: Energy times volume to the 2/3 power equals 2.402 for CO-covered and 2.388 eV nm${}^{2}$ for SCH${}_{3}$-covered nanoparticles (see Table 2).

These differences in excitation energies should in principle be probed by an appropriate experiment. It might be a great challenge to design an optical experiment to distinguish these single-electron levels through the great background of light scattering and absorption. However, recent developments in optical trapping allow for detailed studies of light-matter interaction [43]. In such state-of-the-art optics, one could in principle differentiate between plasmonic and electronic peaks, as the latter will be much narrower. Another possibility for experimental observation of these states would be through tunnelling experiments and other electron-transfer measurements, such as Scanning Tunneling Spectroscopy (STS) or Electron Energy Loss Spectroscopy (EELS). Recent examples of probing single-electron states can be seen in Refs. [16,29,30]. Particularly for the lowest-lying electron states, X-ray techniques might be able to probe them—for instance, X-ray absorption spectroscopy can probe states lying hundreds of eVs below the Fermi level with resolution of 100 meV; see for example [44,45].

If such a method exists, it will allow for quick and reliable characterization of catalysts, as it can provide a direct link between single-electron states and the density of active sites for catalysis. For example, when a Au nanoparticle with a volume of 500 nm${}^{3}$ is covered by thiols, the first excitation energy drops from 18 to 17 meV while the tenth one rises from 60 meV to 64 meV. These small changes in single-elctron energies correspond to a huge change in the density of active sites, which rises from 72 μmol/g for a clean nanoparicle to 273 μmol/g for a thiol-covered one. Probing single-electron states might be a very accurate tool for catalyst characterization.

## 3. Materials and Methods

### 3.1. Atomistic Wulff Construction

The Au nanoparticles used in this study are generated by means of our atomistic-Wulff- construction method. The Wulff theorem states that the equilibrium shape of a crystal is a polyhedron. The distance of each (hkl) face of this polyhedron from the center is proportional to the surface tension of the (hkl) surface of the material [46,47,48].

Wulff construction has been a valuable tool for nanoparticle shapes, and has been used for nanoparticles formed by a variety of materials, including supported Au [34,49], diamond [50], TiO${}_{2}$ [51], Si in amorphous SiO${}_{2}$ [52], diamond in amorphous C [53], Rh and Pd in oxidizing conditions [54], Cu in N gas [55], Au in oxidizing conditions [56], noble metals with an environment [57], complex metal hydrides [58], iron carbides [59], and dawsonites [60,61], just to name a few. The atomistic Wulff construction takes it one step further and fills the Wulff polyhedron with atoms, taking into account the finite size effects at the nanoscale. It has been used successfully in the context of theoretical catalysis for nanoparticles made of Ru [62,63,64], Ag [65], and Au [39,42,61,66,67].

The atomistic models of the nanoparticles used in this work are constructed using the methodology of references [39,42]; for a review of this method, see reference [68]. The stability of these nanoparticles has been checked in a previous work of the authors by means of Molecular Dynamics (MD) simulations using a many-body Effective-Medium Theory (EMT) potential [69].

### 3.2. Numerical Solution of Schrödinger’s Equation

The difficulty in solving the eigenvalue problem defined in Equations (1) and (2) lies in the nature of the potential. In the case of faceted nanoparticles, like those considered here, the potential V(x,y,z) in Equation (2) is a nonseparable one. Furthermore, the eigenstates of the electron must be vanishing in a very difficult boundary condition, *i.e.*, the borders of the nanoparticle.

We overcome these difficulties by using a suitable set of basis functions and expand wave functions on this basis. The choice of the set of the basis functions is crucial, since it has to be a complete and orthonormal set. An extra advantage of a good basis set is that if it is appropriately related to the problem to be solved, good accuracy is achieved with a limited number of basis functions. Here, the basis functions we use are the solutions for the cubic nanoparticle, $\phi_{j}\left(x,y,z\right)$. Each eigenfunction of the system, $\psi_{i}\left(x,y,z\right)$, is expanded as a linear combination of basis functions as follows:

(10) $$\psi_{i}\left(x,y,z\right)=\sum_{j}^{N}c_{i,j}\phi_{j}\left(x,y,z\right)$$

where $c_{i,j}$ is a scalar coefficient describing the weight of the contribution of the $j^{th}$ function of the base to the eigenfunction *i*.

Using a set of basis functions in order to numerically solve the eigenvalue problem, one has firstly to construct the matrix of the Hamiltonian of the system. Then, to diagonalize this matrix, commonly using Linear Algebra PACKage (LAPACK) subroutines in order to get the eigenenergies and the $c_{i,j}$ coefficients.

**Figure 7:**
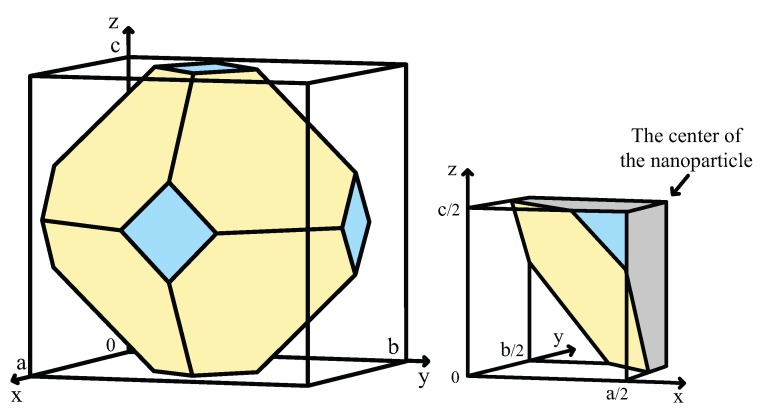
(**Left**): The polyhedron that forms the boundary of the nanoparticle enclosed in the simulated box with dimensions (a,b,c); (**Right**): The octant used to calculate the potential integral. Grey color represents the inner part of the nanoparticle.

We start by finding the bounding box for each nanoparticle. This is a rectangle with dimensions (a,b,c), as shown in Figure 7 (left). We then use the eigenstates of the well-known *“particle-in-a-box"* problem as a set of basis functions:

(11) $$\begin{array}{c} \phi_{n,m,l}\left(x,y,z\right)=\sqrt{\frac{8}{abc}}\sin\left(\frac{n\pi x}{a}\right)\sin\left(\frac{m\pi y}{b}\right)\sin\left(\frac{l\pi z}{c}\right)\\ E_{n,m,l}=\frac{\hbar^{2}\pi^{2}}{2m_{e}}\left(\frac{n^{2}}{a^{2}}+\frac{m^{2}}{b^{2}}+\frac{l^{2}}{c^{2}}\right) \end{array}$$

These are solutions of the Schrödinger equation for the potential energy V(x,y,z) defined as:

(12) $$V\left(x,y,z\right)=\left\{\begin{array}{cc} 0 & 0\leq x\leq a,0\leq y\leq b,\;\text{and}\;0\leq z\leq c\\ \infty & \text{otherwise} \end{array}\right.$$

The advantage of these eigenfunctions is that they are also eigenstates of the kinetic energy operator of our system. We then write each matrix element of the Hamiltonian of the system, $\left(\phi_{n^{\prime },m^{\prime },l^{\prime }},H\phi_{n,m,l}\right)$, as:

(13) $$\begin{array}{ccc} \left(\phi_{n^{\prime },m^{\prime },l^{\prime }},H\phi_{n,m,l}\right) & = & E_{n,m,l}\delta_{n^{\prime },n}\delta_{m^{\prime },m}\delta_{l^{\prime },l}\\ & + & \left(\phi_{n^{\prime },m^{\prime },l^{\prime }},V\left(x,y,z\right)\phi_{n,m,l}\right) \end{array}$$

where $\left(\phi_{n^{\prime },m^{\prime },l^{\prime }},V\left(x,y,z\right)\phi_{n,m,l}\right)$ is the expectation value of the potential energy for the potential defined in Equation (2).

The problem of calculating the $\left(\phi_{n^{\prime },m^{\prime },l^{\prime }},H\phi_{n,m,l}\right)$ is now reduced to calculating the $\left(\phi_{n^{\prime },m^{\prime },l^{\prime }},V\left(x,y,z\right)\phi_{n,m,l}\right)$ integral. We substitute the infinity value of the potential with a large enough value, $V_{0}$. The matrix element then takes the following form:

(14) $$\left(\phi_{n^{\prime },m^{\prime },l^{\prime }},V\left(x,y,z\right)\phi_{n,m,l}\right)=V_{0}\int \phi_{n^{\prime },m^{\prime },l^{\prime }}^{*}\phi_{n,m,l}dV,$$

where the integration is over all volume expept fot the interior of the nanoparticle.

The nonzero integral of V(x,y,z) outside the nanoparticle can be calculated either numerically, using for example a three-dimensional trapezoidal rule, or analytically. The disadvantage of a numerical solution is that it needs a high-density grid in order to be as adaptive as possible to the boundary condition of the potential and also to be able to catch the fluctuations of the functions of the basis. This high-density grid makes the calculation too slowly and not accurately enough.

In this study, both numerical and analytical techniques were performed. In the following, we describe in detail the method used to calculate the expectation value of the potential energy using an analytical integration method.

Due to symmetry, we can use only the region where: $0\leq x\leq \frac{a}{2}$, $0\leq y\leq \frac{b}{2}$, and $0\leq z\leq \frac{c}{2}$, as shown in Figure 7 (right). The integration volume is spilt into a sum of regions. Each region is defined by corner atoms of the nanoparticle and the space between the nanoparticle and the box. After defining these regions, we can solve the potential integral, $V_{region}$, in each of them using the form:

(15) $$V_{region}=\frac{8V_{0}}{abc}\int_{x_{i}}^{x_{f}}\int_{y_{i}}^{y_{f}}\int_{0}^{z_{f}}\sin\left(\frac{n^{\prime }\pi x}{a}\right)\sin\left(\frac{m^{\prime }\pi y}{b}\right)\sin\left(\frac{l^{\prime }\pi z}{c}\right)\sin\left(\frac{n\pi x}{a}\right)\sin\left(\frac{m\pi y}{b}\right)\sin\left(\frac{l\pi z}{c}\right)dzdydx$$

Using the Euler’s formula, we rewrite each sine with exponential functions:

(16) $$\sin\left(x\right)=\frac{e^{ix}-e^{-ix}}{2i}$$

and thus Equation (15) is written as:

(17) $$\begin{array}{ccc} V_{region} & = & \frac{8V_{0}}{abc}\int_{x_{i}}^{x_{f}}\int_{y_{i}}^{y_{f}}\int_{0}^{z_{f}}\left(\frac{e^{\frac{in^{\prime }\pi }{a}x}-e^{-\frac{in^{\prime }\pi }{a}x}}{2i}\right)\left(\frac{e^{\frac{im^{\prime }\pi }{b}y}-e^{-\frac{im^{\prime }\pi }{b}y}}{2i}\right)\left(\frac{e^{\frac{il^{\prime }\pi }{c}z}-e^{-\frac{il^{\prime }\pi }{c}z}}{2i}\right)\\ & \times & \left(\frac{e^{\frac{in\pi }{a}x}-e^{-\frac{in\pi }{a}x}}{2i}\right)\left(\frac{e^{\frac{im\pi }{b}y}-e^{-\frac{im\pi }{b}y}}{2i}\right)\left(\frac{e^{\frac{il\pi }{c}z}-e^{-\frac{il\pi }{c}z}}{2i}\right)dz\;dy\;dx \end{array}$$

Expanding this integral we obtain that $V_{region}$ is given by:

(18) $$\begin{array}{ccc} V_{region} & = & \frac{-8V_{0}}{2^{6}abc}\int_{x_{i}}^{x_{f}}\int_{y_{i}}^{y_{f}}\int_{0}^{z_{f}}(e^{+\frac{i\pi (n^{\prime }+n)}{a}x}e^{+\frac{i\pi (m+m^{\prime })}{b}y}e^{+\frac{i\pi (l+l^{\prime })}{c}z}\;\;\\ & - & e^{+\frac{i\pi (n^{\prime }+n)}{a}x}e^{+\frac{i\pi (m+m^{\prime })}{b}y}e^{+\frac{i\pi (l-l^{\prime })}{c}z}-\;e^{+\frac{i\pi (n^{\prime }+n)}{a}x}e^{+\frac{i\pi (m+m^{\prime })}{b}y}e^{-\frac{i\pi (l-l^{\prime })}{c}z}\;\\ & + & e^{+\frac{i\pi (n^{\prime }+n)}{a}x}e^{+\frac{i\pi (m+m^{\prime })}{b}y}e^{-\frac{i\pi (l+l^{\prime })}{c}z}-\;e^{+\frac{i\pi (n^{\prime }+n)}{a}x}e^{+\frac{i\pi (m-m^{\prime })}{b}y}e^{+\frac{i\pi (l+l^{\prime })}{c}z}\;\\ & + & e^{+\frac{i\pi (n^{\prime }+n)}{a}x}e^{+\frac{i\pi (m-m^{\prime })}{b}y}e^{+\frac{i\pi (l-l^{\prime })}{c}z}+\;e^{+\frac{i\pi (n^{\prime }+n)}{a}x}e^{+\frac{i\pi (m-m^{\prime })}{b}y}e^{-\frac{i\pi (l-l^{\prime })}{c}z}\;\\ & - & e^{+\frac{i\pi (n^{\prime }+n)}{a}x}e^{+\frac{i\pi (m-m^{\prime })}{b}y}e^{-\frac{i\pi (l+l^{\prime })}{c}z}-\;e^{+\frac{i\pi (n^{\prime }+n)}{a}x}e^{-\frac{i\pi (m-m^{\prime })}{b}y}e^{+\frac{i\pi (l+l^{\prime })}{c}z}\;\\ & + & e^{+\frac{i\pi (n^{\prime }+n)}{a}x}e^{-\frac{i\pi (m-m^{\prime })}{b}y}e^{+\frac{i\pi (l-l^{\prime })}{c}z}+\;e^{+\frac{i\pi (n^{\prime }+n)}{a}x}e^{-\frac{i\pi (m-m^{\prime })}{b}y}e^{-\frac{i\pi (l-l^{\prime })}{c}z}\;\\ & - & e^{+\frac{i\pi (n^{\prime }+n)}{a}x}e^{-\frac{i\pi (m-m^{\prime })}{b}y}e^{-\frac{i\pi (l+l^{\prime })}{c}z}+\;e^{+\frac{i\pi (n^{\prime }+n)}{a}x}e^{-\frac{i\pi (m+m^{\prime })}{b}y}e^{+\frac{i\pi (l+l^{\prime })}{c}z}\;\;\\ & - & e^{+\frac{i\pi (n^{\prime }+n)}{a}x}e^{-\frac{i\pi (m+m^{\prime })}{b}y}e^{+\frac{i\pi (l-l^{\prime })}{c}z}-\;e^{+\frac{i\pi (n^{\prime }+n)}{a}x}e^{-\frac{i\pi (m+m^{\prime })}{b}y}e^{-\frac{i\pi (l-l^{\prime })}{c}z}\;\\ & + & e^{+\frac{i\pi (n^{\prime }+n)}{a}x}e^{-\frac{i\pi (m+m^{\prime })}{b}y}e^{-\frac{i\pi (l+l^{\prime })}{c}z}-\;e^{+\frac{i\pi (n^{\prime }-n)}{a}x}e^{+\frac{i\pi (m+m^{\prime })}{b}y}e^{+\frac{i\pi (l+l^{\prime })}{c}z}\;\\ & + & e^{+\frac{i\pi (n^{\prime }-n)}{a}x}e^{+\frac{i\pi (m+m^{\prime })}{b}y}e^{+\frac{i\pi (l-l^{\prime })}{c}z}+\;e^{+\frac{i\pi (n^{\prime }-n)}{a}x}e^{+\frac{i\pi (m+m^{\prime })}{b}y}e^{-\frac{i\pi (l-l^{\prime })}{c}z}\;\\ & - & e^{+\frac{i\pi (n^{\prime }-n)}{a}x}e^{+\frac{i\pi (m+m^{\prime })}{b}y}e^{-\frac{i\pi (l+l^{\prime })}{c}z}+\;e^{+\frac{i\pi (n^{\prime }-n)}{a}x}e^{+\frac{i\pi (m-m^{\prime })}{b}y}e^{+\frac{i\pi (l+l^{\prime })}{c}z}\;\\ & - & e^{+\frac{i\pi (n^{\prime }-n)}{a}x}e^{+\frac{i\pi (m-m^{\prime })}{b}y}e^{+\frac{i\pi (l-l^{\prime })}{c}z}-\;e^{+\frac{i\pi (n^{\prime }-n)}{a}x}e^{+\frac{i\pi (m-m^{\prime })}{b}y}e^{-\frac{i\pi (l-l^{\prime })}{c}z}\;\\ & + & e^{+\frac{i\pi (n^{\prime }-n)}{a}x}e^{+\frac{i\pi (m-m^{\prime })}{b}y}e^{-\frac{i\pi (l+l^{\prime })}{c}z}+\;e^{+\frac{i\pi (n^{\prime }-n)}{a}x}e^{-\frac{i\pi (m-m^{\prime })}{b}y}e^{+\frac{i\pi (l+l^{\prime })}{c}z}\;\\ & - & e^{+\frac{i\pi (n^{\prime }-n)}{a}x}e^{-\frac{i\pi (m-m^{\prime })}{b}y}e^{+\frac{i\pi (l-l^{\prime })}{c}z}-\;e^{+\frac{i\pi (n^{\prime }-n)}{a}x}e^{-\frac{i\pi (m-m^{\prime })}{b}y}e^{-\frac{i\pi (l-l^{\prime })}{c}z}\;\\ & + & e^{+\frac{i\pi (n^{\prime }-n)}{a}x}e^{-\frac{i\pi (m-m^{\prime })}{b}y}e^{-\frac{i\pi (l+l^{\prime })}{c}z}-\;e^{+\frac{i\pi (n^{\prime }-n)}{a}x}e^{-\frac{i\pi (m+m^{\prime })}{b}y}e^{+\frac{i\pi (l+l^{\prime })}{c}z}\;\\ & + & e^{+\frac{i\pi (n^{\prime }-n)}{a}x}e^{-\frac{i\pi (m+m^{\prime })}{b}y}e^{+\frac{i\pi (l-l^{\prime })}{c}z}+\;e^{+\frac{i\pi (n^{\prime }-n)}{a}x}e^{-\frac{i\pi (m+m^{\prime })}{b}y}e^{-\frac{i\pi (l-l^{\prime })}{c}z}\;\\ & - & e^{+\frac{i\pi (n^{\prime }-n)}{a}x}e^{-\frac{i\pi (m+m^{\prime })}{b}y}e^{-\frac{i\pi (l+l^{\prime })}{c}z}-\;e^{-\frac{i\pi (n^{\prime }-n)}{a}x}e^{+\frac{i\pi (m+m^{\prime })}{b}y}e^{+\frac{i\pi (l+l^{\prime })}{c}z}\;\\ & + & e^{-\frac{i\pi (n^{\prime }-n)}{a}x}e^{+\frac{i\pi (m+m^{\prime })}{b}y}e^{+\frac{i\pi (l-l^{\prime })}{c}z}+\;e^{-\frac{i\pi (n^{\prime }-n)}{a}x}e^{+\frac{i\pi (m+m^{\prime })}{b}y}e^{-\frac{i\pi (l-l^{\prime })}{c}z}\;\\ & - & e^{-\frac{i\pi (n^{\prime }-n)}{a}x}e^{+\frac{i\pi (m+m^{\prime })}{b}y}e^{-\frac{i\pi (l+l^{\prime })}{c}z}+\;e^{-\frac{i\pi (n^{\prime }-n)}{a}x}e^{+\frac{i\pi (m-m^{\prime })}{b}y}e^{+\frac{i\pi (l+l^{\prime })}{c}z}\;\\ & - & e^{-\frac{i\pi (n^{\prime }-n)}{a}x}e^{+\frac{i\pi (m-m^{\prime })}{b}y}e^{+\frac{i\pi (l-l^{\prime })}{c}z}-\;e^{-\frac{i\pi (n^{\prime }-n)}{a}x}e^{+\frac{i\pi (m-m^{\prime })}{b}y}e^{-\frac{i\pi (l-l^{\prime })}{c}z}\;\\ & + & e^{-\frac{i\pi (n^{\prime }-n)}{a}x}e^{+\frac{i\pi (m-m^{\prime })}{b}y}e^{-\frac{i\pi (l+l^{\prime })}{c}z}+\;e^{-\frac{i\pi (n^{\prime }-n)}{a}x}e^{-\frac{i\pi (m-m^{\prime })}{b}y}e^{+\frac{i\pi (l+l^{\prime })}{c}z}\;\\ & - & e^{-\frac{i\pi (n^{\prime }-n)}{a}x}e^{-\frac{i\pi (m-m^{\prime })}{b}y}e^{+\frac{i\pi (l-l^{\prime })}{c}z}-\;e^{-\frac{i\pi (n^{\prime }-n)}{a}x}e^{-\frac{i\pi (m-m^{\prime })}{b}y}e^{-\frac{i\pi (l-l^{\prime })}{c}z}\;\\ & + & e^{-\frac{i\pi (n^{\prime }-n)}{a}x}e^{-\frac{i\pi (m-m^{\prime })}{b}y}e^{-\frac{i\pi (l+l^{\prime })}{c}z}-\;e^{-\frac{i\pi (n^{\prime }-n)}{a}x}e^{-\frac{i\pi (m+m^{\prime })}{b}y}e^{+\frac{i\pi (l+l^{\prime })}{c}z}\;\\ & + & e^{-\frac{i\pi (n^{\prime }-n)}{a}x}e^{-\frac{i\pi (m+m^{\prime })}{b}y}e^{+\frac{i\pi (l-l^{\prime })}{c}z}+\;e^{-\frac{i\pi (n^{\prime }-n)}{a}x}e^{-\frac{i\pi (m+m^{\prime })}{b}y}e^{-\frac{i\pi (l-l^{\prime })}{c}z}\;\\ & - & e^{-\frac{i\pi (n^{\prime }-n)}{a}x}e^{-\frac{i\pi (m+m^{\prime })}{b}y}e^{-\frac{i\pi (l+l^{\prime })}{c}z}+\;e^{-\frac{i\pi (n^{\prime }+n)}{a}x}e^{+\frac{i\pi (m+m^{\prime })}{b}y}e^{+\frac{i\pi (l+l^{\prime })}{c}z}\;\\ & - & e^{-\frac{i\pi (n^{\prime }+n)}{a}x}e^{+\frac{i\pi (m+m^{\prime })}{b}y}e^{+\frac{i\pi (l-l^{\prime })}{c}z}-\;e^{-\frac{i\pi (n^{\prime }+n)}{a}x}e^{+\frac{i\pi (m+m^{\prime })}{b}y}e^{-\frac{i\pi (l-l^{\prime })}{c}z}\;\\ & + & e^{-\frac{i\pi (n^{\prime }+n)}{a}x}e^{+\frac{i\pi (m+m^{\prime })}{b}y}e^{-\frac{i\pi (l+l^{\prime })}{c}z}-\;e^{-\frac{i\pi (n^{\prime }+n)}{a}x}e^{+\frac{i\pi (m-m^{\prime })}{b}y}e^{+\frac{i\pi (l+l^{\prime })}{c}z}\;\\ & + & e^{-\frac{i\pi (n^{\prime }+n)}{a}x}e^{+\frac{i\pi (m-m^{\prime })}{b}y}e^{+\frac{i\pi (l-l^{\prime })}{c}z}+\;e^{-\frac{i\pi (n^{\prime }+n)}{a}x}e^{+\frac{i\pi (m-m^{\prime })}{b}y}e^{-\frac{i\pi (l-l^{\prime })}{c}z}\;\\ & - & e^{-\frac{i\pi (n^{\prime }+n)}{a}x}e^{+\frac{i\pi (m-m^{\prime })}{b}y}e^{-\frac{i\pi (l+l^{\prime })}{c}z}-\;e^{-\frac{i\pi (n^{\prime }+n)}{a}x}e^{-\frac{i\pi (m-m^{\prime })}{b}y}e^{+\frac{i\pi (l+l^{\prime })}{c}z}\;\\ & + & e^{-\frac{i\pi (n^{\prime }+n)}{a}x}e^{-\frac{i\pi (m-m^{\prime })}{b}y}e^{+\frac{i\pi (l-l^{\prime })}{c}z}+\;e^{-\frac{i\pi (n^{\prime }+n)}{a}x}e^{-\frac{i\pi (m-m^{\prime })}{b}y}e^{-\frac{i\pi (l-l^{\prime })}{c}z}\;\\ & - & e^{-\frac{i\pi (n^{\prime }+n)}{a}x}e^{-\frac{i\pi (m-m^{\prime })}{b}y}e^{-\frac{i\pi (l+l^{\prime })}{c}z}+\;e^{-\frac{i\pi (n^{\prime }+n)}{a}x}e^{-\frac{i\pi (m+m^{\prime })}{b}y}e^{+\frac{i\pi (l+l^{\prime })}{c}z}\;\\ & - & e^{-\frac{i\pi (n^{\prime }+n)}{a}x}e^{-\frac{i\pi (m+m^{\prime })}{b}y}e^{+\frac{i\pi (l-l^{\prime })}{c}z}-\;e^{-\frac{i\pi (n^{\prime }+n)}{a}x}e^{-\frac{i\pi (m+m^{\prime })}{b}y}e^{-\frac{i\pi (l-l^{\prime })}{c}z}\;\\ & + & e^{-\frac{i\pi (n^{\prime }+n)}{a}x}e^{-\frac{i\pi (m+m^{\prime })}{b}y}e^{-\frac{i\pi (l+l^{\prime })}{c}z})\;dz\;dy\;dx \end{array}$$

The above integral can be reduced using the fact that its 64 terms are all of the form:

(19) $$s\left(t\right)e^{\frac{i\pi N(t)}{a}x}e^{\frac{i\pi M(t)}{b}y}e^{\frac{i\pi L(t)}{c}z}$$

where s(t) is the sign of each term; and N(t), M(t), and L(t) represent each one of the 64 sets of combinations of ($\pm n^{\prime },\pm n),\left(\pm m^{\prime },\pm m\right)$, and $(\pm l^{\prime },\pm l$). Substituting N(t), M(t) and L(t) into $V_{region}$ and taking into account that the box used is a cubic one (a=b=c), Equation (18) can be written as a sum over 64 terms in form:

(20) $$V_{region}=\frac{-8V_{0}}{2^{6}a^{3}}\sum_{t=1}^{64}s\left(t\right)\int_{x_{i}}^{x_{f}}\int_{y_{i}}^{y_{f}}\int_{0}^{z_{f}}e^{\frac{i\pi N(t)}{a}x}e^{+\frac{i\pi M(t)}{a}y}e^{+\frac{i\pi L(t)}{a}z}dz\;dy\;dx$$

With limits defined by the borders of each region given as: $x_{i}<x<x_{f}$, the lines, $y_{i}=a_{i}x+b_{i}<y<y_{f}=a_{f}x+b_{f}$, and the surfaces $z_{i}=0<z<z_{f}=\frac{d-hx-ky}{l}$, where $d=\sqrt{h^{2}+k^{2}+l^{2}}d_{hkl}$. This triple integral of each one of these terms can be calculated analytically and is given by:

(21) $$\begin{array}{ccc} & & \int_{x_{i}}^{x_{f}}\int_{y_{i}}^{y_{f}}\int_{0}^{z_{f}}e^{\frac{i\pi N(t)}{a}x}e^{+\frac{i\pi M(t)}{a}y}e^{+\frac{i\pi L(t)}{a}z}dz\;dy\;dx\\ & = & \frac{-2a^{3}l^{2}}{\pi^{3}L\left(t\right)\left[M\left(t\right)l-L\left(t\right)k\right]\left[N\left(t\right)l-L\left(t\right)h+\left[M\left(t\right)l-L\left(t\right)k\right]a_{f}\right]}\{\\ & & \;\sin[\frac{\pi d}{al}L\left(t\right)+\frac{\pi b_{f}}{al}\left[M\left(t\right)l-L\left(t\right)k\right]+\frac{\pi x_{f}}{al}\left[N\left(t\right)l-L\left(t\right)h+\left[M\left(t\right)l-L\left(t\right)k\right]a_{f}\right]]\\ & & \;-\sin[\frac{\pi d}{al}L\left(t\right)+\frac{\pi b_{f}}{al}\left[M\left(t\right)l-L\left(t\right)k\right]+\frac{\pi x_{i}}{al}\left[N\left(t\right)l-L\left(t\right)h+\left[M\left(t\right)l-L\left(t\right)k\right]a_{f}\right]]\}\\ & + & \frac{2a^{3}l^{2}}{\pi^{3}L\left(t\right)\left[M\left(t\right)l-L\left(t\right)k\right]\left[N\left(t\right)l-L\left(t\right)h+\left[M\left(t\right)l-L\left(t\right)k\right]a_{i}\right]}\{\\ & & \;\sin[\frac{\pi d}{al}L\left(t\right)+\frac{\pi b_{i}}{al}\left[M\left(t\right)l-L\left(t\right)k\right]+\frac{\pi x_{f}}{al}\left[N\left(t\right)l-L\left(t\right)h+\left[M\left(t\right)l-L\left(t\right)k\right]a_{i}\right]]\\ & & \;-\sin[\frac{\pi d}{al}L\left(t\right)+\frac{\pi b_{i}}{al}\left[M\left(t\right)l-L\left(t\right)k\right]+\frac{\pi x_{i}}{al}\left[N\left(t\right)l-L\left(t\right)h+\left[M\left(t\right)l-L\left(t\right)k\right]a_{i}\right]]\}\\ & + & \frac{2a^{3}}{\pi^{3}L\left(t\right)M\left(t\right)\left[N\left(t\right)+a_{f}M\left(t\right)\right]}\{\\ & & \;\sin\left[\frac{\pi b_{f}}{a}M\left(t\right)+\frac{\pi x_{f}}{a}\left[N\left(t\right)+a_{f}M\left(t\right)\right]\right]-sin\left[\frac{\pi b_{f}}{a}M\left(t\right)+\frac{\pi x_{i}}{a}\left[N\left(t\right)+a_{f}M\left(t\right)\right]\right]\}\\ & + & \frac{2a^{3}}{\pi^{3}L\left(t\right)M\left(t\right)\left[N\left(t\right)+a_{i}M\left(t\right)\right]}\{\\ & & \;\sin\left[\frac{\pi b_{i}}{a}M\left(t\right)+\frac{\pi x_{f}}{a}\left[N\left(t\right)+a_{i}M\left(t\right)\right]\right]-sin\left[\frac{\pi b_{i}}{a}M\left(t\right)+\frac{\pi x_{i}}{a}\left[N\left(t\right)+a_{i}M\left(t\right)\right]\right]\} \end{array}$$

There are a few cases where one or more of the denominators of Equation (21) are equal to zero. For example, when L(t)=0, all of the denominators are equal to zero. In such a case, it is easy to show that the nominator of the same fraction also equals zero and thus the fraction takes the indeterminate form of 0/0. This indeterminate form can be addressed either by applying the l’Hopital rule, or (more easily) by defining the integral again without the terms that are equal to zero and solving it from scratch. In the code this is implemented using *if* statements, where each one uses a formula to return the value of the integral for each specific case. Equation (21) was used in order to calculate the $V_{region}$ of each one of the regions.

Finally, the expectation value for the potential energy is given by the sum of $V_{region}$ over all the regions as:

(22) $$\left(\phi_{n^{\prime },m^{\prime },l^{\prime }},V\left(x,y,z\right)\phi_{n,m,l}\right)=\sum_{regions}V_{region}$$

With the expectation values of the kinetic and the potential energy known, we construct the Hamiltonian matrix. This matrix is a real symmetric one and can be diagonalized using the TRED2 and TQL2 subroutines from the Fortran LAPACK library. The former reduces the real symmetric matrix of the Hamiltonian to a symmetric tridiagonal one, and the latter finds the eigenvalues and eigenfunctions of the reduced matrix. These eigenvalues and eigenfunctions are the eigenenergies and eigenstates of a free electron in the nanoparticle.

## 4. Conclusions

We presented eigenenergies and eigenstates as well as the density of states for several distinct shapes of gold nanoparticles. We find that both the absolute values of eigenenergies and their degeneracies depend crucially on the shape of the nanoparticle. The density of states clearly shows a distinguishable shape-dependent electronic structure. The first excitation energy alone can be used as a safe criterion for the determination of the shape of Au nanoparticles, which in turn can yield the density of active sites for catalysis. We hope that this work might be the first step towards the identification of nanoparticle shapes using single-electron levels. As such, it might be a step towards catalyst design as an extra characterization tool for catalysts by means of the energy levels of their electrons.

## Figures and Tables

**Table 1:** Comparison of calculated, $E_{i}^{Calc}$, and analytically obtained values, $E_{i}$, of the first thirty eigenenergies of a spherical nanoparticle with volume *V* = 481.42 nm${}^{3}$.

*i*	$E_{i}^{calc}$ (meV)	$E_{i}$ (meV)	*i*	$E_{i}^{calc}$ (meV)	$E_{i}$ (meV)	*i*	$E_{i}^{calc}$ (meV)	$E_{i}$ (meV)
1	16.577	15.907	11	81.605	78.704	21	111.900	107.914
2	33.690	32.543	12	81.693	78.704	22	112.031	107.914
3	33.953	32.543	13	81.791	78.704	23	112.122	107.914
4	33.953	32.543	14	81.791	78.704	24	112.122	107.914
5	55.534	53.538	15	82.017	78.704	25	112.259	107.914
6	55.534	53.538	16	82.343	78.704	26	112.404	107.914
7	55.652	53.538	17	82.343	78.704	27	112.404	107.914
8	55.842	53.538	18	99.605	96.189	28	112.851	107.914
9	56.070	53.538	19	100.370	96.189	29	113.054	107.914
10	66.307	63.630	20	100.370	96.189	30	138.328	133.323

**Table 2:** Energy eigenvalues for five different nanoparticle shapes. The scaled energy, $\Delta E_{i}=\left(E_{i}\;-\;E_{0}\right)V^{\frac{2}{3}}$ is shown, where $E_{i}$ is the *i*-th energy eigenvalue and *V* is te volume of each nanoparticle.

*i*	$\Delta E_{i}$ (eV nm${}^{2}$)				
	Sphere	CO	SCH${}_{3}$	Clean	Cube
0	0.000	0.000	0.000	0.000	0.000
1	1.022	1.068	1.089	1.105	1.128
2	1.022	1.068	1.089	1.105	1.128
3	1.022	1.069	1.089	1.105	1.128
4	2.312	2.402	2.388	2.183	2.256
5	2.312	2.402	2.389	2.183	2.256
6	2.312	2.402	2.517	2.721	2.256
7	2.312	2.446	2.517	2.721	3.008
8	2.312	2.446	2.517	2.721	3.008
9	2.931	3.071	3.120	3.030	3.008
10	3.857	3.981	4.041	3.774	3.384

## Abbreviations

The following abbreviations are used in this manuscript:

DFT: Density-functional Theory
DOS: Density of states

## References

[1] Sun Y., Xia Y. (2002). Shape-Controlled Synthesis of Gold and Silver Nanoparticles. Science.

[2] Grzelczak M., Pérez-Juste J., Mulvaney P., Liz-Marzán L. (2008). Shape control in gold nanoparticle synthesis. Chem. Soc. Rev..

[3] Mostafa S., Behafarid F., Croy J.R., Ono L.K., Li L., Yang J.C., Frenkel A.I., Cuenya B.R. (2010). Shape-Dependent Catalytic Properties of Pt Nanoparticles. J. Am. Chem. Soc..

[4] Kovács G., Fodor S., Vulpoi A., Schrantz K., Dombi A., Hernádi K., Danciu V., Pap Z., Baia L. (2015). Polyhedral Pt *vs.* spherical Pt nanoparticles on commercial titanias: Is shape tailoring a guarantee of achieving high activity?. J. Catal..

[5] Vajda K., Saszet K., Kedves E., Kása Z., Danciu V., Baia L., Magyari K., Hernádi K., Kovács G., Pap Z. (2016). Shape-controlled agglomeration of TiO_2_ nanoparticles. New insights on polycrystallinity *vs.* single crystals in photocatalysis. Ceram. Int..

[6] Remediakis I.N., Lopez N., Nørskov J.K. (2005). CO Oxidation on Rutile-Supported Au Nanoparticles. Angew. Chem. Int. Ed..

[7] Barnard A.S., Lin X.M., Curtiss L.A. (2005). Equilibrium Morphology of Face-Centered Cubic Gold Nanoparticles >3 nm and the Shape Changes Induced by Temperature. J. Phys. Chem. B.

[8] Fasi A., Palinko I., Hernadi K., Kiricsi I. (2006). Formation of Au nanorods and nanoforks over MgO support. React. Kinet. Catal. Lett..

[9] Ahmadi T.S., Wang Z.L., Green T.C., Henglein A., Sayed M.A.E. (2007). Shape-Controlled Synthesis of Colloidal Platinum Nanoparticles. Science.

[10] Ruffino F., Bongiorno C., Giannazzo F., Roccaforte F., Raineri V., Grimaldi M. (2007). Effect of surrounding environment on atomic structure and equilibrium shape of growing nanocrystals: Gold in/on SiO_2_. Nanoscale Res. Lett..

[11] Kittel C. (2005). Introduction to Solid State Physics.

[12] Van Aert S., Batenburg K.J., Rossell M.D., Erni R., Van Tendeloo G. (2011). Three-dimensional atomic imaging of crystalline nanoparticles. Nature.

[13] Sivaramakrishnan S., Wen J., Scarpelli M.E., Pierce B.J., Zuo J.M. (2010). Equilibrium shapes and triple line energy of epitaxial gold nanocrystals supported on TiO_2_(110). Phys. Rev. B.

[14] De Heer W.A. (1993). The physics of simple metal clusters: Experimental aspects and simple models. Rev. Mod. Phys..

[15] Walter M., Akola J., Lopez-Acevedo O., Jadzinsky P.D., Calero G., Ackerson C.J., Whetten R.L., Grönbeck H., Häkkinen H. (2008). A unified view of ligand-protected gold clusters as superatom complexes. Proc. Natl. Acad. Sci. USA.

[16] Stiehler C., Calaza F., Schneider W.D., Nilius N., Freund H.J. (2015). Molecular Adsorption Changes the Quantum Structure of Oxide-Supported Gold Nanoparticles: Chemisorption versus Physisorption. Phys. Rev. Lett..

[17] Pelton M., Aizpurua J., Bryant G. (2008). Metal-nanoparticle plasmonics. Laser Photonics Rev..

[18] West P., Ishii S., Naik G., Emani N., Shalaev V., Boltasseva A. (2010). Searching for better plasmonic materials. Laser Photonics Rev..

[19] Mie G. (1908). Beiträge zur Optik trüber Medien, speziell kolloidaler Metallösungen. Ann. Phys..

[20] Maier S.A. (2007). Plasmonics: Fundamentals and Applications.

[21] Klimov V.I., Mikhailovski A.A., Xu S., Malko A., Hollingsworth J.A., Leatherdale C.A., Eisler H.J., Bawendi M.G. (2000). Optical Gain and Stimulated Emission in Nanocrystal Quantum Dots. Science.

[22] Lal S., Link S., Halas N.J. (2007). Nano-optics from sensing to waveguiding. Nat. Photonics.

[23] Kim S.K., Day R.W., Cahoon J.F., Kempa T.J., Song K.D., Park H.G., Lieber C.M. (2012). Tuning Light Absorption in Core/Shell Silicon Nanowire Photovoltaic Devices through Morphological Design. Nano Lett..

[24] Beqa L., Singh A.K., Khan S.A., Senapati D., Arumugam S.R., Ray P.C. (2011). Gold Nanoparticle-Based Simple Colorimetric and Ultrasensitive Dynamic Light Scattering Assay for the Selective Detection of Pb(II) from Paints, Plastics, and Water Samples. ACS Appl. Mater. Interfaces.

[25] Hourahine B., Papoff F. (2012). The geometrical nature of optical resonances: From a sphere to fused dimer nanoparticles. Meas. Sci. Technol..

[26] Hourahine B., Papoff F. (2013). Optical control of scattering, absorption and lineshape in nanoparticles. Opt. Express.

[27] Von Delft J., Ralph D. (2001). Spectroscopy of discrete energy levels in ultrasmall metallic grains. Phys. Rep..

[28] Bolotin K.I., Kuemmeth F., Pasupathy A.N., Ralph D.C. (2004). Metal-nanoparticle single-electron transistors fabricated using electromigration. Appl. Phys. Lett..

[29] Kuemmeth F., Bolotin K.I., Shi S.F., Ralph D.C. (2008). Measurement of Discrete Energy-Level Spectra in Individual Chemically Synthesized Gold Nanoparticles. Nano Lett..

[30] Lin X., Nilius N., Freund H.J., Walter M., Frondelius P., Honkala K., Häkkinen H. (2009). Quantum Well States in Two-Dimensional Gold Clusters on MgO Thin Films. Phys. Rev. Lett..

[31] Kac M. (1966). Can One Hear the Shape of a Drum?. Am. Math. Mon..

[32] Hearing the Shape of a Drum. https://en.wikipedia.org/wiki/Hearing_the_shape_of_a_drum.

[33] Tolea F., Tolea M. (2015). Hearing shapes of few electrons quantum drums: A configuration-interaction study. Physica B.

[34] Lopez N., Nørskov J., Janssens T., Carlsson A., Puig-Molina A., Clausen B., Grunwaldt J.D. (2004). The adhesion and shape of nanosized Au particles in a Au/TiO_2_ catalyst. J. Catal..

[35] Remediakis I.N., Lopez N., Nørskov J.K. (2005). CO oxidation on gold nanoparticles: Theoretical studies. Appl. Catal. A Gen..

[36] Quintana M., Ke X., Tendeloo G.V., Meneghetti M., Bittencourt C., Prato M. (2010). Light-Induced Selective Deposition of Au Nanoparticles on Single-Wall Carbon Nanotubes. ACS Nano.

[37] Ueda K., Kawasaki T., Hasegawa H., Tanji T., Ichihashi M. (2008). First observation of dynamic shape changes of a gold nanoparticle catalyst under reaction gas environment by transmission electron microscopy. Surf. Interface Anal..

[38] Häkkinen H., Walter M., Grönbeck H. (2006). Divide and Protect: Capping Gold Nanoclusters with Molecular Gold-Thiolate Rings. J. Phys. Chem. B.

[39] Barmparis G.D., Remediakis I.N. (2012). Dependence on CO adsorption of the shapes of multifaceted gold nanoparticles: A density functional theory. Phys. Rev. B.

[40] Bittencourt C., Felten A., Douhard B., Colomer J.F., Tendeloo G.V., Drube W., Ghijsen J., Pireaux J.J. (2007). Metallic nanoparticles on plasma treated carbon nanotubes: Nano^2^hybrids. Surf. Sci..

[41] Espinosa E., Ionescu R., Bittencourt C., Felten A., Erni R., Tendeloo G.V., Pireaux J.J., Llobet E. (2007). Metal-decorated multi-wall carbon nanotubes for low temperature gas sensing. Thin Solid Films.

[42] Barmparis G.D., Honkala K., Remediakis I.N. (2013). Thiolate adsorption on Au(*hkl*) and equilibrium shape of large thiolate-covered gold nanoparticles. J. Chem. Phys..

[43] Marago O.M., Jones P.H., Gucciardi P.G., Volpe G., Ferrari A.C. (2013). Optical trapping and manipulation of nanostructures. Nat. Nano.

[44] Plummer E. (1991). Electronic states and phases of K_*x*_C_60_ from photoemission and X-ray absorption spectroscopy. Nature.

[45] Liu W., Hu E., Jiang H., Xiang Y., Weng Z., Li M., Fan Q., Yu X., Altman E.I., Wang H. (2016). A highly active and stable hydrogen evolution catalyst based on pyrite-structured cobalt phosphosulfide. Nat. Commun..

[46] Wulff G. (1901). Zur Frage der Geschwindigkeit des Wachstums und der Auflösung der Krystallflächen. Z. Kristallogr..

[47] Herring C. (1951). Some Theorems on the Free Energies of Crystal Surfaces. Phys. Rev..

[48] Rosakis P. (2014). Continuum Surface Energy from a Lattice Model. Netw. Heterog. Media.

[49] Molina L.M., Hammer B. (2003). Active Role of Oxide Support during CO Oxidation at Au/MgO. Phys. Rev. Lett..

[50] Barnard A., Zapol P. (2004). A model for the phase stability of arbitrary nanoparticles as a function of size and shape. J. Chem. Phys..

[51] Barnard A.S., Curtiss L.A. (2005). Prediction of TiO_2_ Nanoparticle Phase and Shape Transitions Controlled by Surface Chemistry. Nano Lett..

[52] Hadjisavvas G., Remediakis I.N., Kelires P.C. (2006). Insights into the Shape and Faceting of Embedded Si/*α*-SiO_2_ Nanocrystals. Phys. Rev. B.

[53] Kopidakis G., Remediakis I.N., Fyta M.G., Kelires P.C. (2007). Atomic and electronic structure of crystalline-amorphous carbon interfaces. Diam. Relat. Mater..

[54] Mittendorfer F., Seriani N., Dubay O., Kresse G. (2007). Morphology of mesoscopic Rh and Pd nanoparticles under oxidizing conditions. Phys. Rev. B.

[55] Soon A., Wong L., Delley B., Stampfl C. (2008). Morphology of copper nanoparticles in a nitrogen atmosphere: A first-principles investigation. Phys. Rev. B.

[56] Shi H., Stampfl C. (2008). Shape and surface structure of gold nanoparticles under oxidizing conditions. Phys. Rev. B.

[57] Cortes-Huerto R., Goniakowski J., Noguera C. (2013). An efficient many-body potential for the interaction of transition and noble metal nano-objects with an environment. J. Chem. Phys..

[58] Kim K.C., Dai B., Johnson J.K., Sholl D.S. (2009). Assessing nanoparticle size effects on metal hydride thermodynamics using the Wulff construction. Nanotechnology.

[59] Pham T.H., Duan X., Qian G., Zhou X., Chen D. (2014). CO Activation Pathways of Fischer Tropsch Synthesis on *χ*-Fe_5_C_2_ (510): Direct versus Hydrogen-Assisted CO Dissociation. J. Phys. Chem. C.

[60] Lodziana Z., Stoica G., Perez-Ramirez J. (2011). Reevaluation of the Structure and Fundamental Physical Properties of Dawsonites by DFT Studies. Inorg. Chem..

[61] Honkala K., Lodziana Z., Remediakis I.N., Lopez N. (2014). Expanding and Reducing Complexity in Materials Science Models with Relevance in Catalysis and Energy. Top. Catal..

[62] Honkala K., Hellman A., Remediakis I.N., Logadottir A., Carlsson A., Dahl S., Christensen C., Nørskov J.K. (2005). Ammonia synthesis from first-principles calculations. Science.

[63] Hellman A., Honkala K., Remediakis I.N., Logadottir A., Carlsson A., Dahl S., Christensen C.H., Nørskov J.K. (2006). Insights into ammonia synthesis from first-principles. Surf. Sci..

[64] Hellman A., Honkala K., Remediakis I.N., Logadottir A., Carlsson A., Dahl S., Christensen C.H., Nørskov J.K. (2009). Ammonia synthesis and decomposition on a Ru-based catalyst modeled by first-principles. Surf. Sci..

[65] Vile G., Baudouin D., Remediakis I.N., Coperet C., Lopez N., Perez-Ramirez J. (2013). Silver Nanoparticles for Olefin Production: New Insights into the Mechanistic Description of Propyne Hydrogenation. ChemCatChem.

[66] Gómez-Graña S., Goris B., Altantzis T., Fernández-López C., Carbó-Argibay E., Guerrero-Martínez A., Almora-Barrios N., López N., Pastoriza-Santos I., Pérez-Juste J. (2013). Au@Ag nanoparticles: Halides stabilize 100 facets. J. Phys. Chem. Lett..

[67] Almora-Barrios N., Novell-Leruth G., Whiting P., Liz-Marzán L., López N. (2014). Theoretical description of the role of halides, silver, and surfactants on the structure of gold nanorods. Nano Lett..

[68] Barmparis G.D., Lodziana Z., Lopez N., Remediakis I.N. (2015). Nanoparticle shapes by using Wulff constructions and first-principles calculations. Beilstein J. Nanotechnol..

[69] Barmparis G.D., Maniadaki A.E., Kopidakis G., Remediakis I.N. (2016). Wulff construction and molecular dynamics simulations for Au nanoparticles. J. Chem. Eng. Chem. Res..

